# Supplementary material for: Hearing Health Awareness and the Need for Educational Outreach Amongst Teachers in Malawi
Source: Audiol Res. 2023 Apr 12;13(2):271–84. doi: 10.3390/audiolres13020024 (PMC10135795; doi:10.3390/audiolres13020024)
Supplement: Supplementary file 1 [file audiolres-13-00024-s001.zip › audiolres-2212033-supplementary.pdf]

Listen Up!
SAVE THE EARS

Hello!

We are ABC Hearing Clinic and Training Center with

Grant Kapalamula & Memory Khomera

You can find us:
hearingabcclinic@gmail.com

0888211091

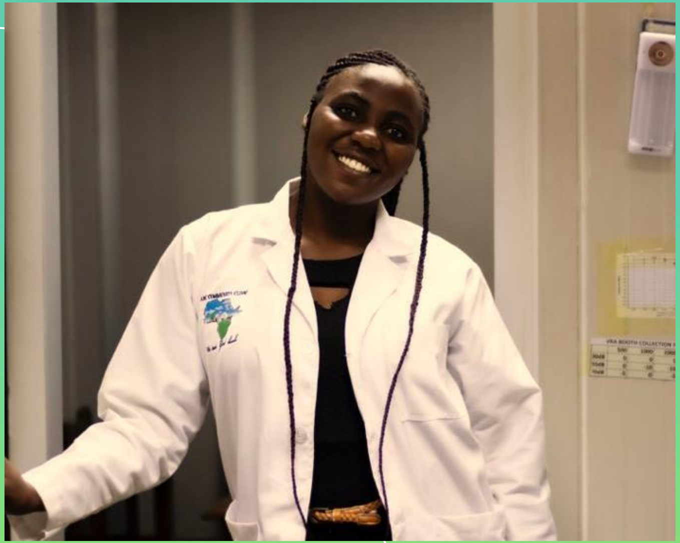

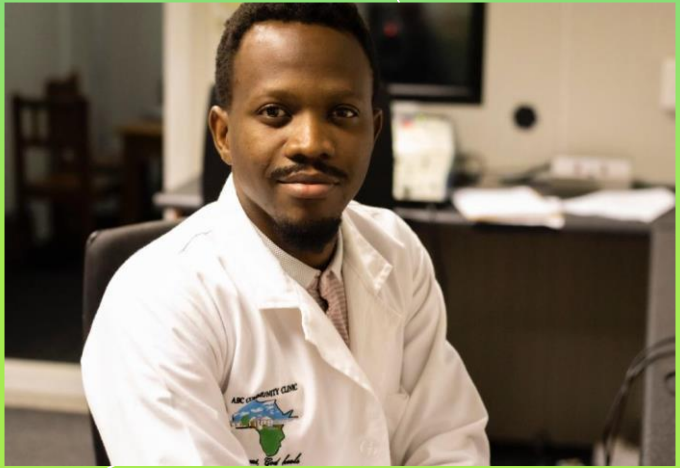

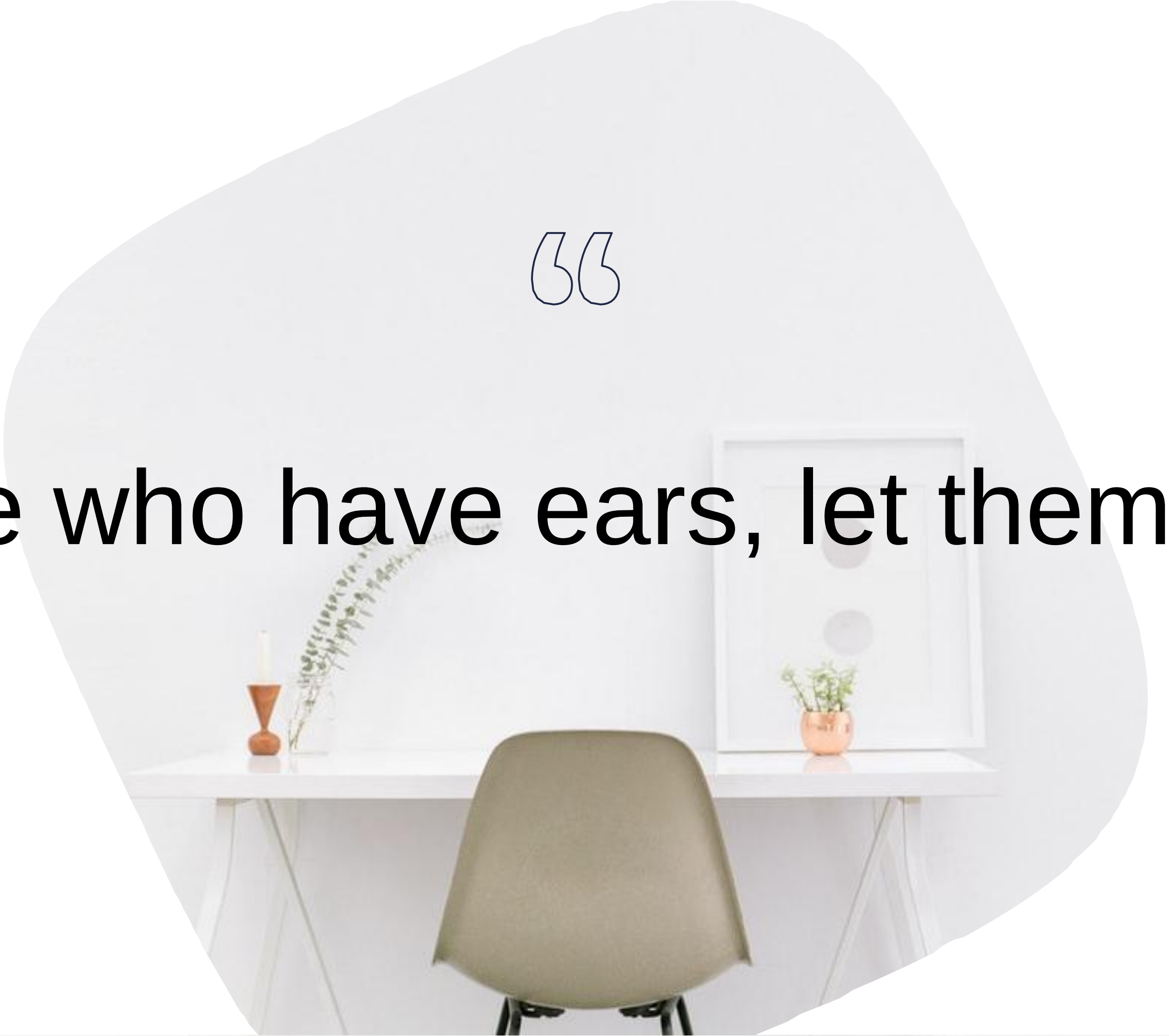

“

Those who have ears, let them hear

Overview

- How we Hear?
- Hearing Loss
- Importance of Hearing
- How to take care of Ears
- **Signs and Symptoms of Hearing Loss**
- Management and Referral

1

How We Hear?

Let’s talk senses

Basics about Ears

Outer Ear

Pinna

Ear Canal

Middle Ear

Ear Drum

Middle Ear Bones – Hammer, Anvil & Stirrup

Inner Ear

Cochlea

Auditory Nerves

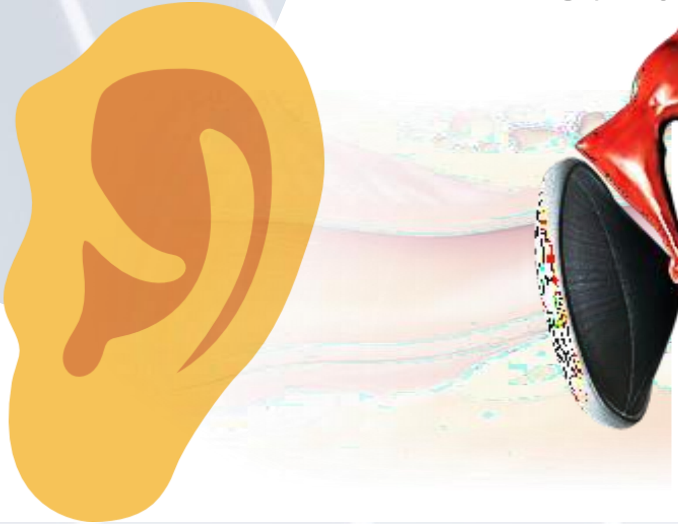

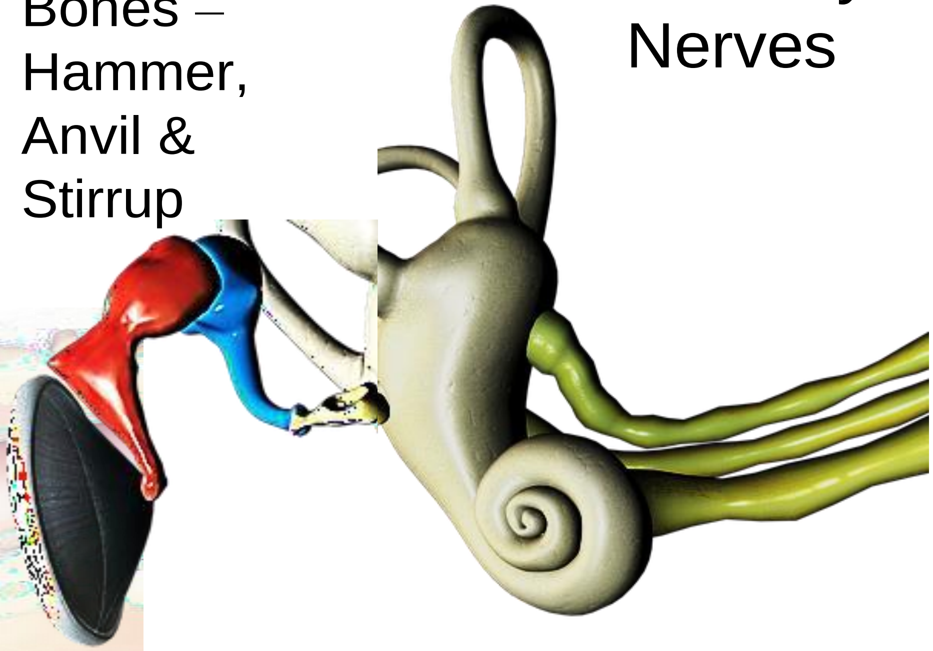

How we hear

- First – Sound enters the ear to the ear drum
- Second – Sound goes through the ear drum & middle ear bones
- Third – Sound enters the inner ear to the brain

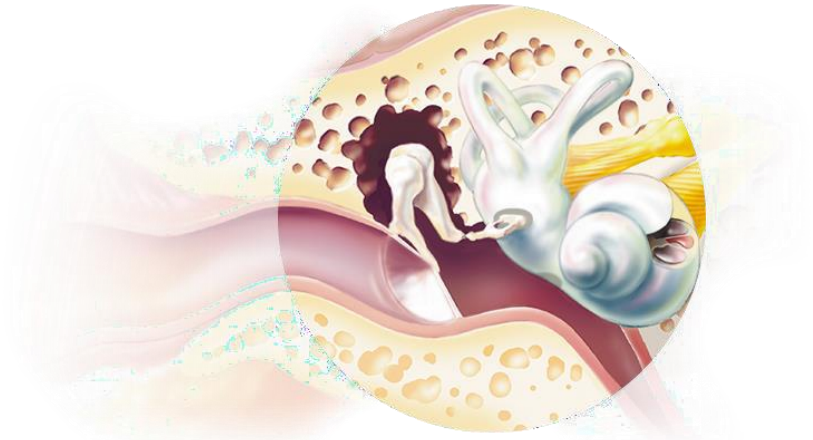

2 Hearing Loss

What is hearing loss?

- Hearing loss is when a person cannot hear well and so has a problem talking and hearing what people say.
- There are different levels of hearing difficulty

Sound clips by NIOSH

Normal Hearing

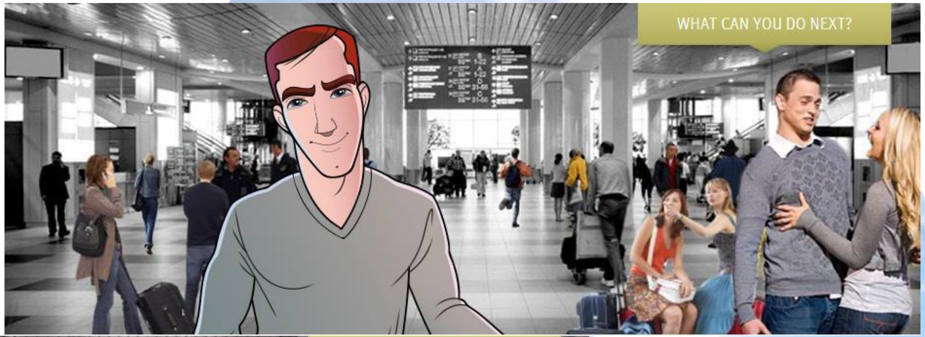

Mild Hearing Loss

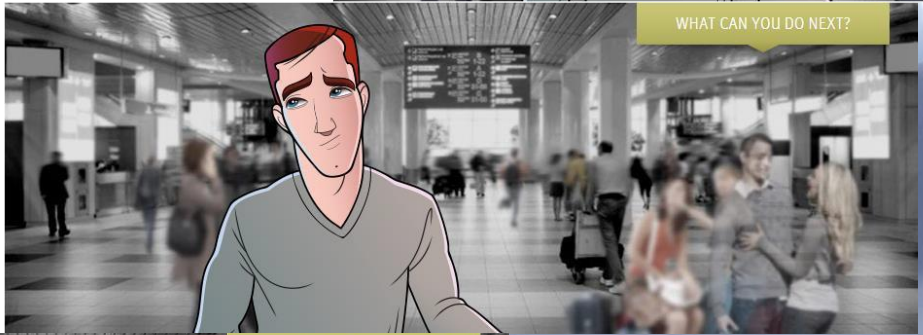

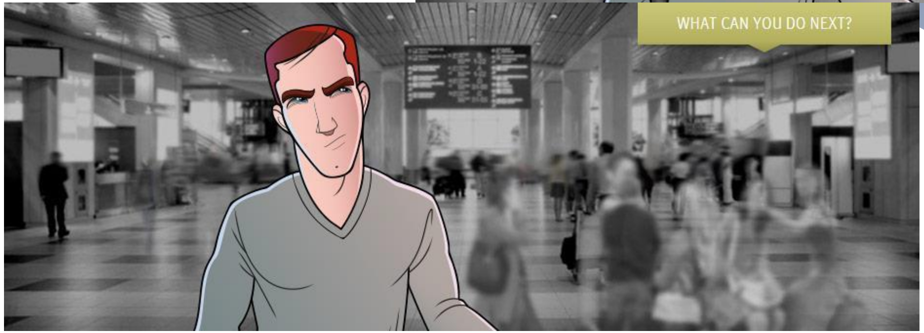

Moderate Hearing Loss

Courtesy of Hear The World

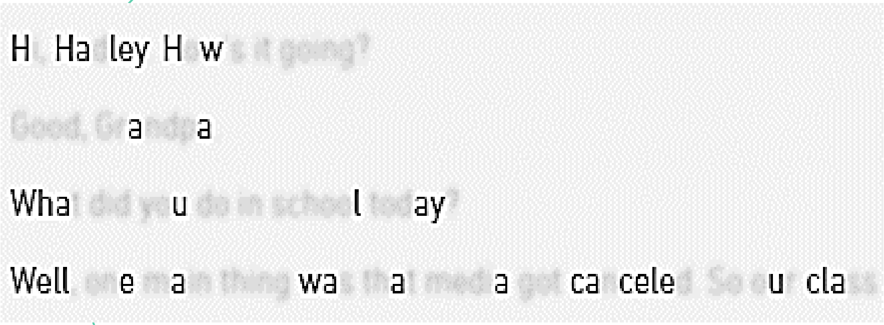

Severe Hearing Loss

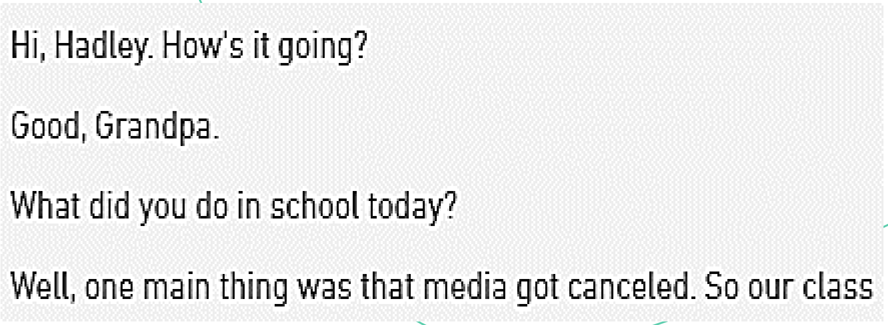

Normal Conversation

Courtesy of Starkey

Where the sound is not going through also counts as classification

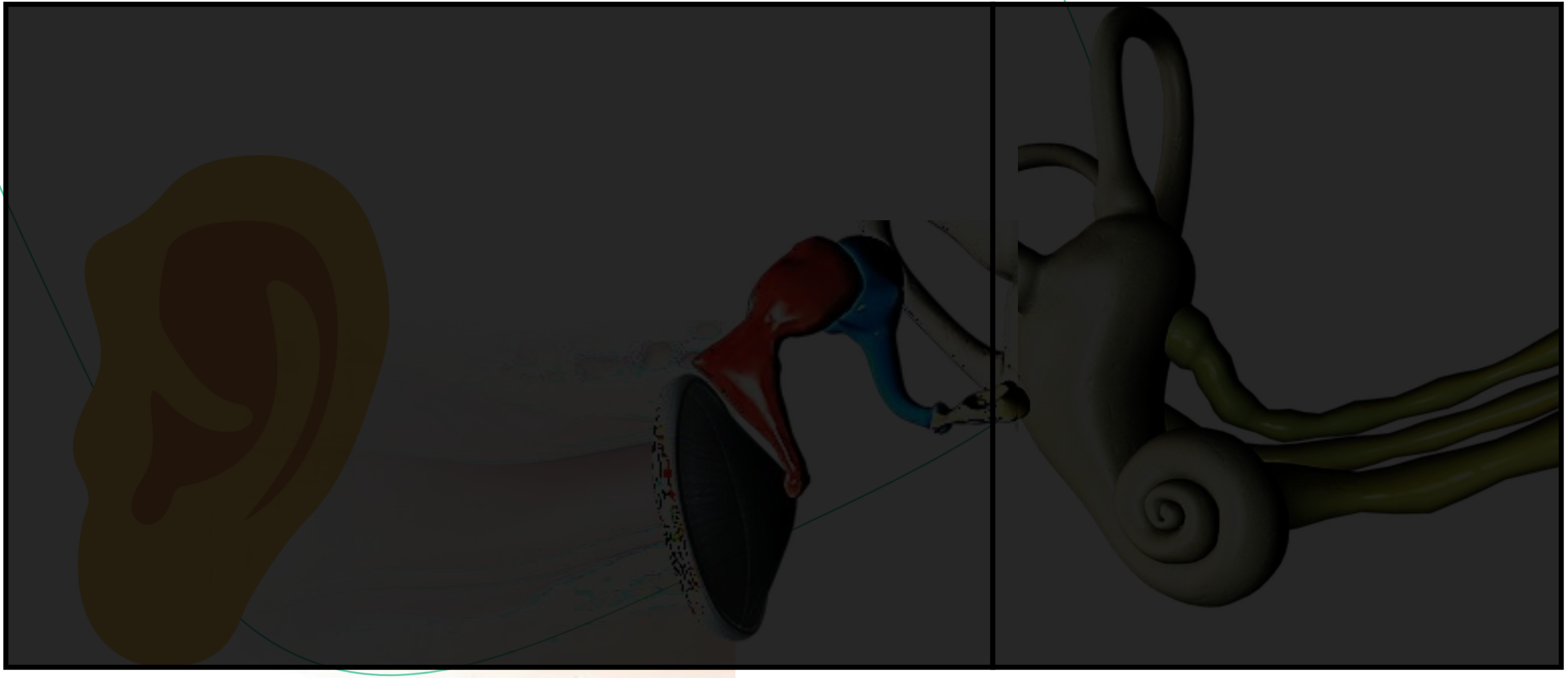

Type: Conductive

Type: Sensory

Causes of Hearing Loss

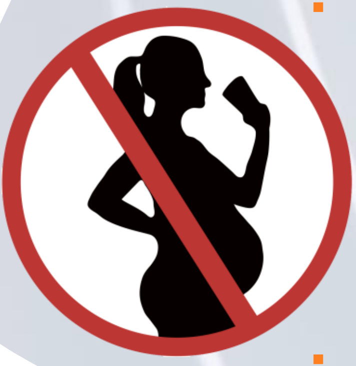

- Drugs
  - Anti-Malaria/some anti-biotics
  - recreational
- Complicated Pregnancies
- Complicated Birth
  - Illness after birth
  - lack of oxygen

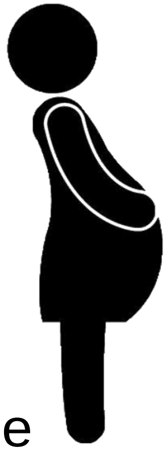

Before Birth

- Inheritance
- Prematurity or Low birth-weight
- Severe Jaundice (Yellow Baby)
- Infections during pregnancy
  - Rubella/German Measles
  - Syphilis

Causes of Hearing Loss

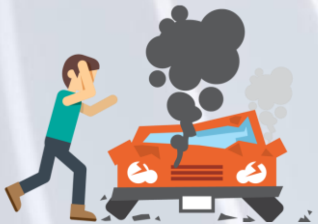

- Accidents
- Excessive Wax
- Glue Ear (Flu/Cold)

After Birth

- Childhood diseases
  - Measles/Mumps/Meningitis
- Ear Infections
- Drugs
  - Anti-malaria/some anti-biotics
- Noise Exposure
  - Maize mill
  - Head sets

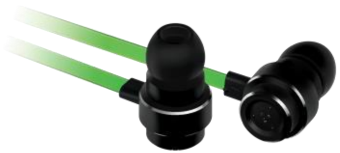

3 Importance of Hearing

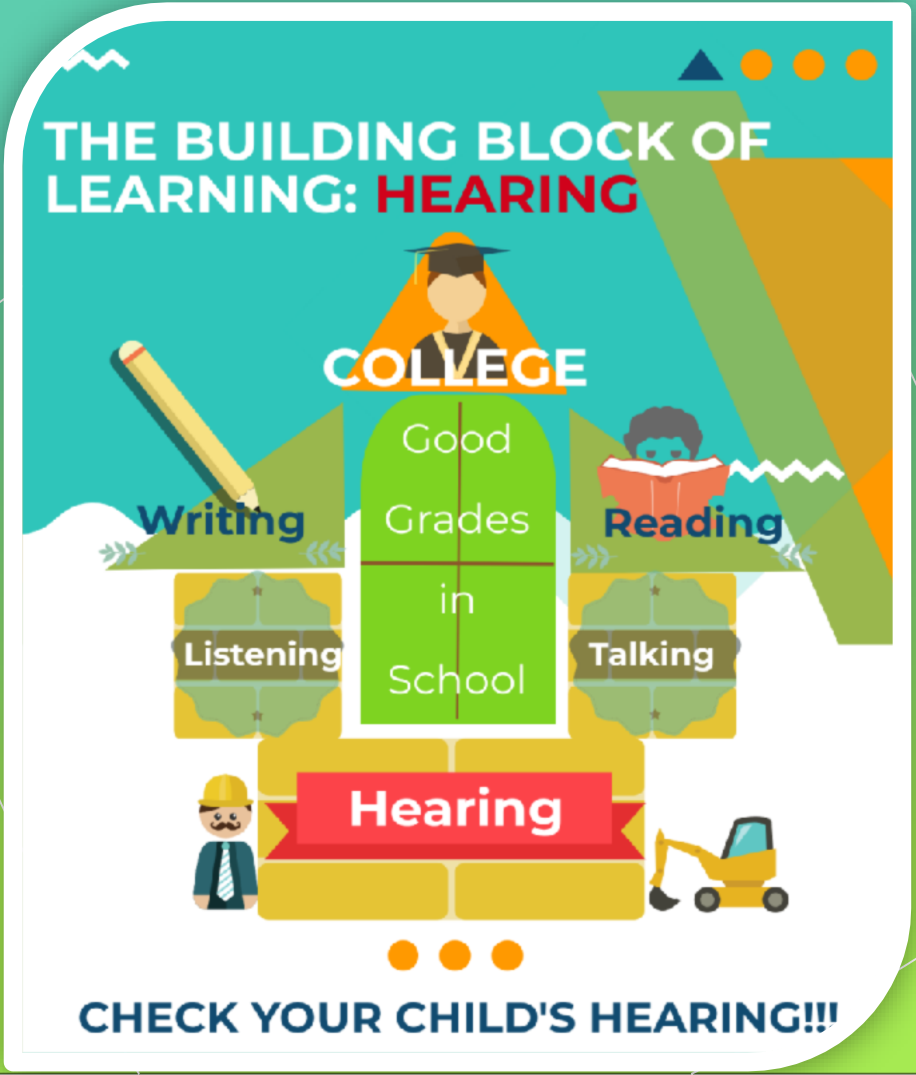
THE BUILDING BLOCK OF LEARNING: HEARING
COLLEGE
Good Grades in School
Writing
Reading
Listening
Talking
Hearing
CHECK YOUR CHILD'S HEARING!!!

Impact of Hearing Loss on a Child

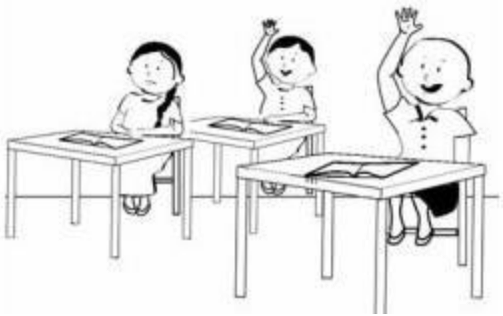

Lack of participation in class

Slower progress in education

Poor/no speech

Difficulty getting independent

Socially Isolated from playing with peers

Lack of communication/expression may lead to social problems

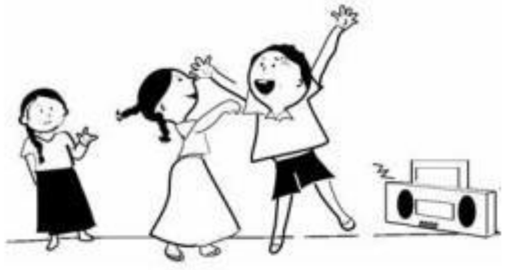

WHO fig 1&2

Impact of Hearing Loss

1. Social Isolation
2. Strain on relationships because of lack of communication
3. Reduced safety awareness (Work or Home)
4. Difficulty getting or maintaining a job
5. Unable to communicate needs and so reduced quality of life
6. Lack of speech if deaf from childhood

4 Signs & Symptoms of Hearing Loss

Signs of Hearing Loss in Class

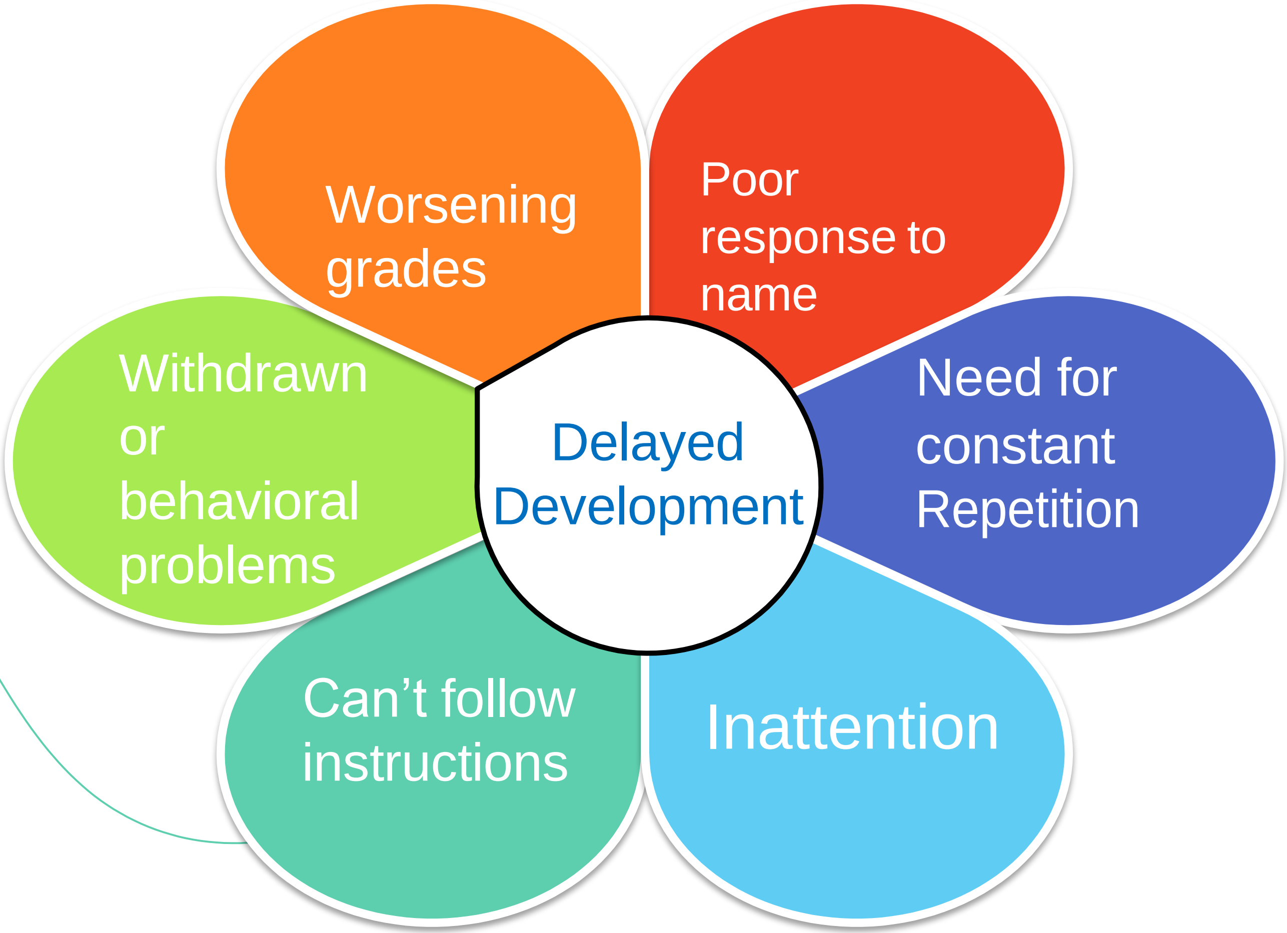

Hearing Loss Statistics

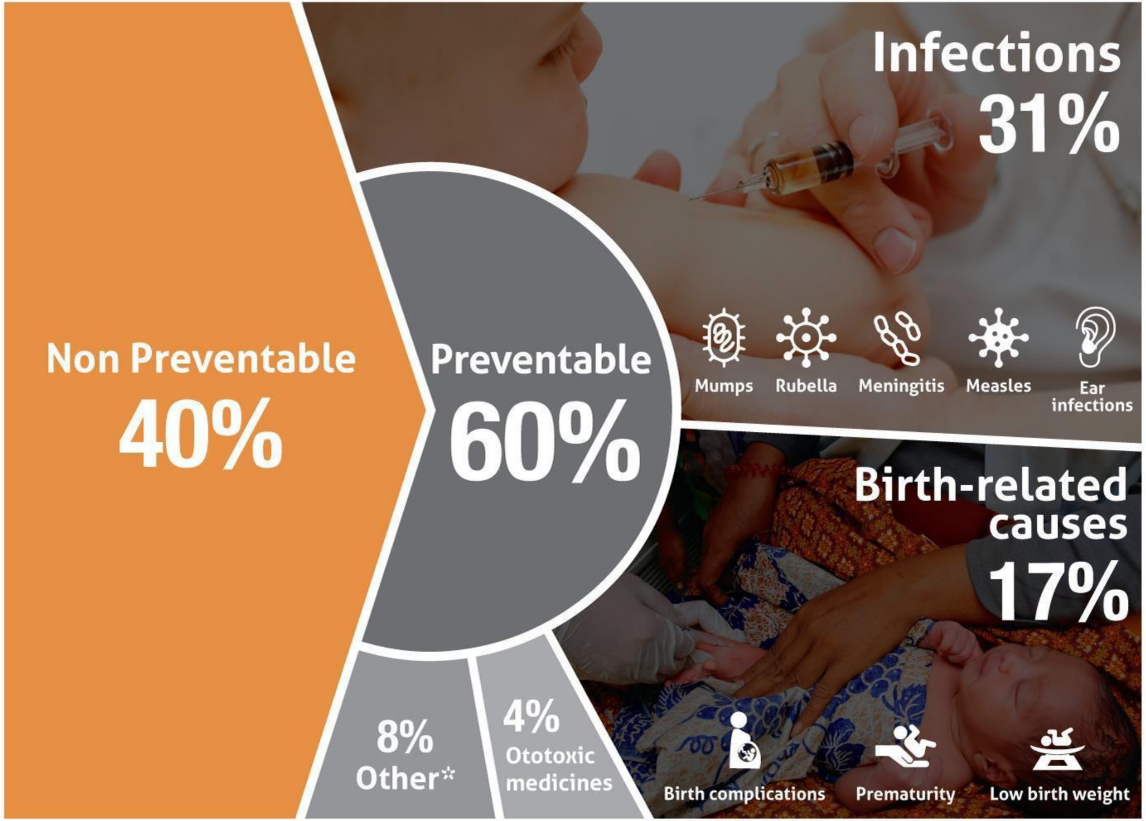

WHO fig 3

as much as 11 in 100 children could have a **preventable** hearing loss in Malawi!

Mulwafu, et al., 2019

5

Management & Referral

How do you manage hearing loss?

How to avoid hearing loss

- Do not insert dirty fingers or objects
- Do not swim in dirty water

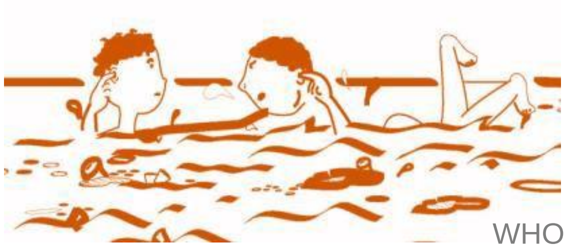

WHO 4 & 5

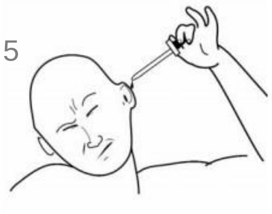

- Do not put anything in the ears – oil, herbal remedies
- Do not clean ears with cotton buds/matchsticks/sticks
  - Wax is good until it's excessive
- Avoid head trauma
  - Wear helmets, seatbelts

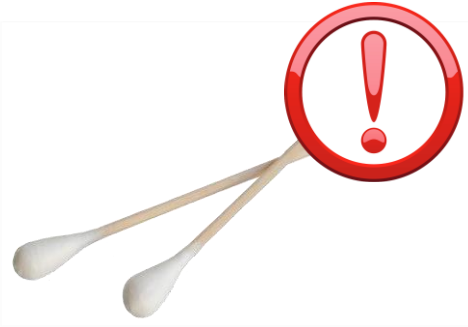

How to avoid hearing loss

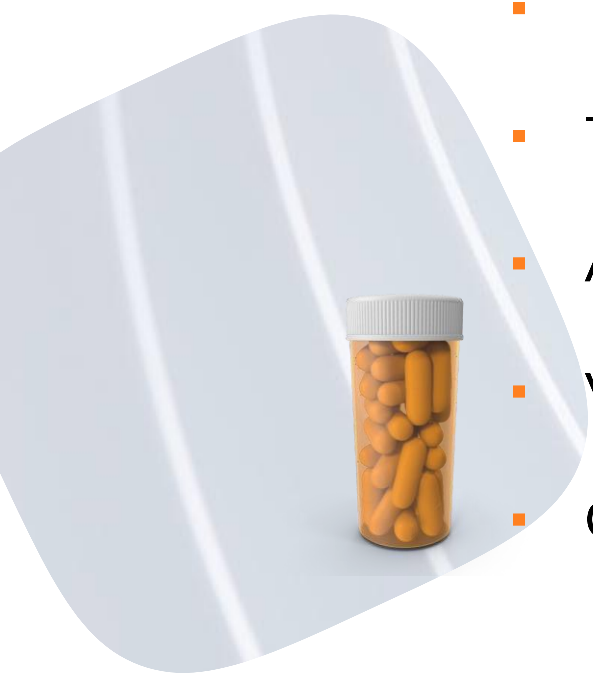

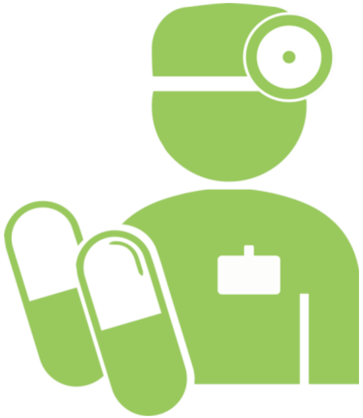

- Treat jaundice
- Treat infections ASAP
- Avoid certain drugs/ only take prescriptions
- Vaccinate and treat pregnant women
- Good ante-natal care

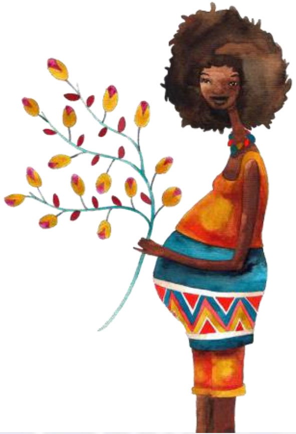

Avoid excessive noise

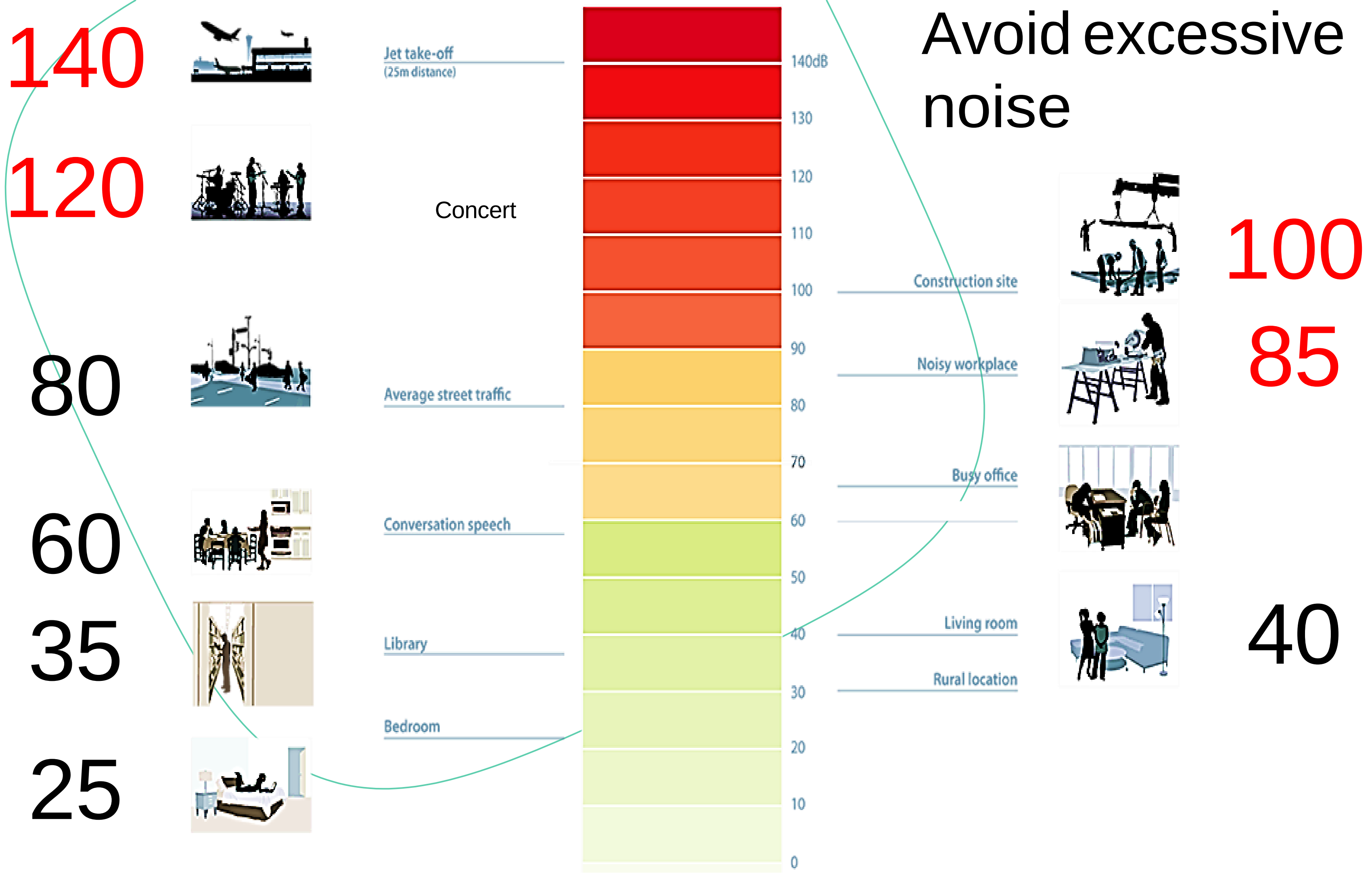

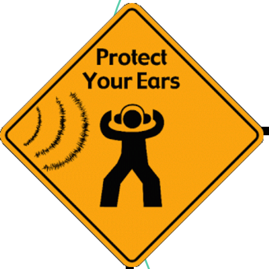

The Recommended Noise Intensity/Loudness to Duration Exposures according to OSHA

Intensity	85	90	95	100	105	110	115
Duration	8 hours	4 hours	2 hours	1 hour	30 mins	15 mins	7:30 mins

Every 5dB increase halves the time that is “safe” to listen to

Infection Management

- Symptoms
  - Fever
  - Sore Ear
  - Runny Ear (Pus)
  - Long lasting colds in children
- Management
  - Refer to a Doctor
  - Dry mop (wipe)

Infection Dry Mopping

WASH HANDS!!

1

2

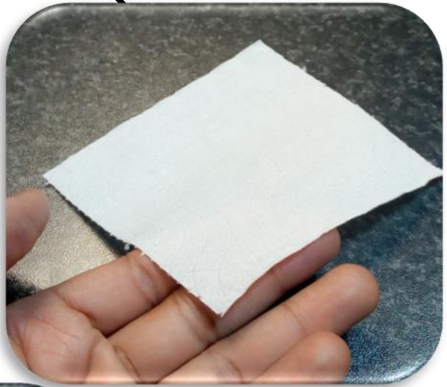

3

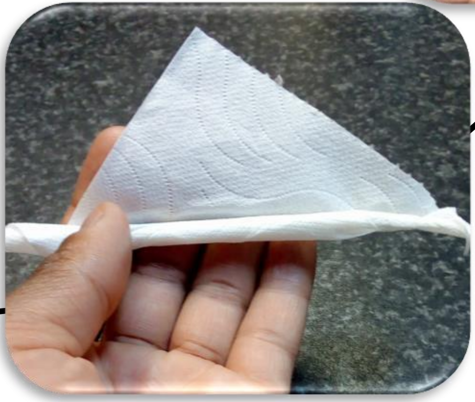

4

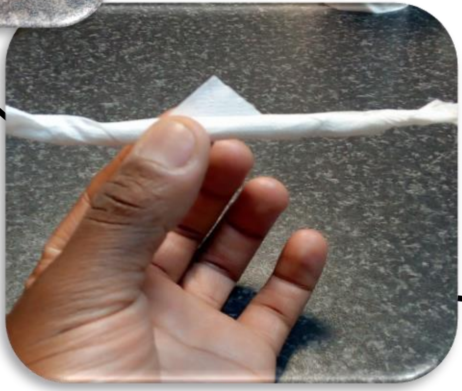

5

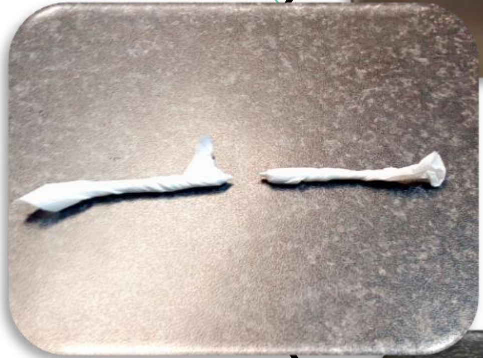

6

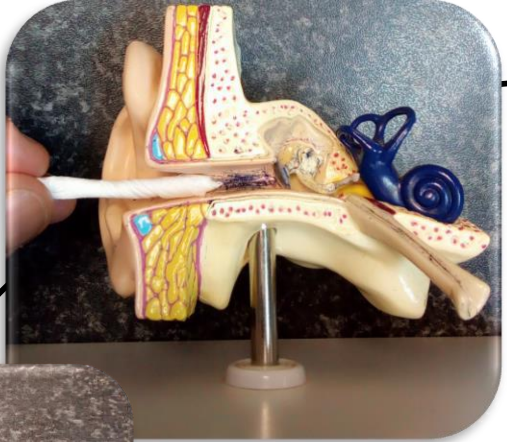

How to manage Hearing Loss

- Refer to Audiologists
  - Listening Devices
  - Clean the ears
  - Manage infections
- Communication Strategies
  - Manage sitting plan
  - Get the attention of the child
  - Speak louder
  - Encourage questions
    - “Can you repeat?”

Listening Devices Include

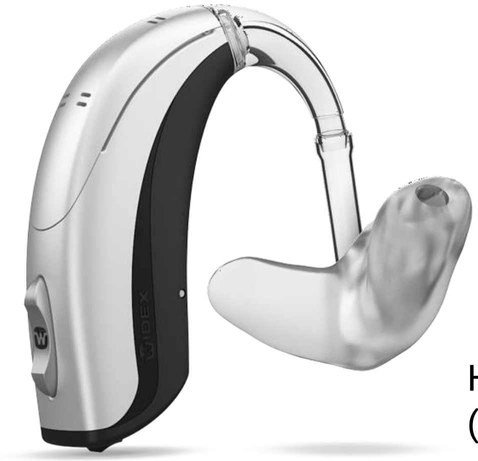

Hearing Aid
(mold)

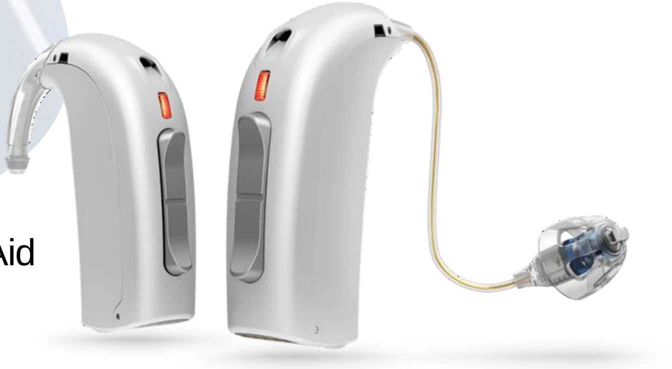

Hearing Aid
(dome)

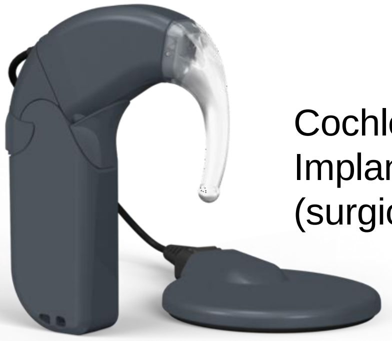

Cochlear
Implant
(surgical)

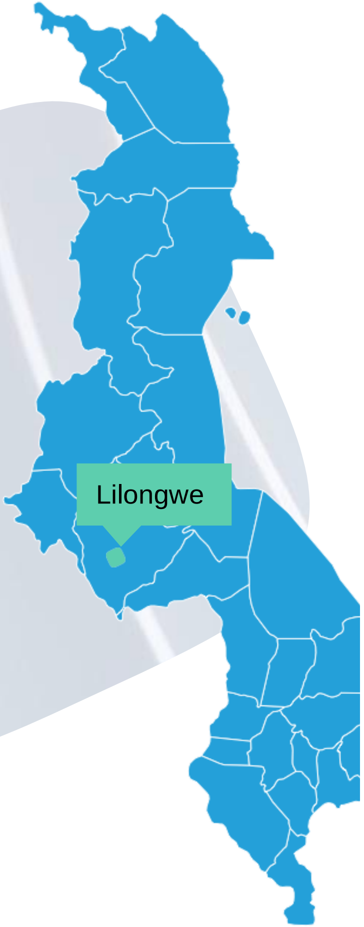

Location

ABC HEARING CLINIC & TRAINING CENTRE

a ministry of EARS Incorporated, Australia

ABC Clinic, Kaunda Road, Area 47, P.O. Box 161, Lilongwe.

hearingabcclinic@gmail.com, +265 888 211 091

Let's review some concepts

How we Hear?

Hearing Loss

Importance of Hearing

How to take care of Ears

Signs and Symptoms

Manage and Refer

References

- WHO. (2006). Primary Ear and Hearing Care Training Resource: Basic Level. *World Health Organization Chronic Disease Prevention and Management, 1-23.*
- WHO. (2006). Primary Ear and Hearing Care Training Resource: Basic Level. *World Health Organization Chronic Disease Prevention and Management, 1-110.*
- WHO. (2016). Childhood Hearing Loss: Strategies for Prevention and Care. *World Health Organization.*
- Starkey Hearing Loss Simulator (Conversation) at https://www.starkey.com/hearing-loss-simulator/simulator.
- Hear The World Foundation Simulator (What Hearing Loss looks like) at https://www.hear-the-world.com/en/knowledge/hearing-loss/what-hearing-loss-sounds-like.
- CDC NIOSH Sound Clips at https://www.cdc.gov/niosh/mining/content/hlsoundslike.html
- Occupational Safety and Health Administration. (2008, June 23). *Occupational Noise Exposure*. Retrieved from Occupational Safety and Health Administration: https://www.osha.gov/SLTC/noisehearingconservation.html
- Mulwafu, et al. (2019). Children with Hearing Loss in Malawi, a Cohort Study. *Bulletin of the World Health Organization, 97:654-662.*
- Presentation template by SlidesCarnival
- Images by cleanpng.com
